# Influence of thermal tempering processes on color characteristics of different monolithic computer-assisted design and computer-assisted manufacturing ceramic materials

**DOI:** 10.4317/jced.55869

**Published:** 2019-07-01

**Authors:** Niwut Juntavee, Pithiwat Uasuwan

**Affiliations:** 1 Department of Prosthodontics, Faculty of Dentistry, Khon Kaen University, Khon Kaen, Thailand; 2 Division of Biomaterials and Prosthodontics Research, Faculty of Dentistry, Khon Kaen University, Khon Kaen, Thailand

## Abstract

**Background:**

The optical properties of dental restoration were influenced by the sintering parameters. This study investigated the effects of different tempering processes on optical properties of three monolithic Cad-Cam ceramics.

**Material and Methods:**

135 monolithic material bars (4 mm width, 14 mm length, 1.2 mm thickness) were prepared from yttria-stabilized tetragonal zirconia polycrystalline (inCoris TZI, I), zirconia-reinforced lithium silicate (Vita Suprinity, V), and lithium disilicate glass (e.max CAD, E) ceramics, with different tempering processes through slow (S), normal (N), and fast (F) cooling (n=15). The color appearance (∆EW), translucency parameter (TP), contrast ratio (CR), and opalescence parameter (OP) were determined. ANOVA and Bonferroni’s multiple comparisons were determined for significant difference (α=0.05). The grain sizes were microscopically examined by scanning electron microscope. The phase transformation of zirconia was determined using X ray diffraction.

**Results:**

The mean±sd of ΔEW, TP, CR, OP were 74.15±0.46, 1.26±0.15, 0.977±0.006, 1.02±0.12 for IS; 74.00±0.83, 1.27±0.19, 0.977±0.007, 1.02±0.12 for IN; 74.44±0.64, 1.70±0.08, 0.965±0.003, 1.30±0.07 for IF; 73.35±1.32, 2.44±0.24, 0.958±0.006, 2.10±0.20 for VS; 66.37±0.88, 4.05±0.3, 0.911±0.010, 3.18±0.20 for VN; 67.02±0.65, 3.79±0.17, 0.919±0.006, 3.01±0.13 for VF; 60.01±0.30, 5.53±0.17, 0.821±0.006, 2.71±0.06 for ES; 60.18±0.23, 5.49±0.17, 0.822±0.006, 2.66±0.05 for EN; and 59.82±0.26, 5.36±0.06, 0.826±0.002, 2.64±0.07 for EF. The color parameters were significantly affected by type of materials, tempering processes, and their interactions (*p*<0.05). Phase transformation from t→m related with tempering procedure for zirconia.

**Conclusions:**

Rapid thermal tempering process of Y-TZP resulted in larger grain size and t→m phase transformation leading to higher translucency. To achieve optimum translucency, a fast thermal tempering process was suggested for inCoris TZI and IPS e.max CAD, whilst a normal tempering process was recommended for Vita Suprinity.

** Key words:**Color, cooling process, contrast, opalescence, thermal tempering, translucency.

## Introduction

An increase in esthetic demands and technological drive has led to the implementation of various materials with excellent physical properties (1). All ceramic restorations are considered the materials of choice to fulfill patients’ demands for esthetics and biosafety as well as being metal-free (2). Several new ceramic materials have been developed; for instance, glass ceramics (lithium disilicate, zirconia-reinforced lithium silicate), hybrid ceramic, and zirconia. Lithium disilicate glass-ceramics with good mechanical and excellent optical properties are commonly used in single and short span dental restorations, especially in the esthetic zone (3,4). In order to sufficiently enhance strength to withstand occlusal force, zirconia-reinforced lithium silicate glass-ceramics have been developed by adding 10% zirconia to glassy content. Currently, zirconia are used for restorative dentistry such as posterior crowns, bridges, and implant components because of its extraordinary mechanical properties together with its transformation toughening mechanism activated by stress induction (5,6). Zirconia is mainly composed of zero glassy content and three crystalline structural phases – monoclinic (m), tetragonal (t), and cubic (c). The m-phase is stable below 1170ºC but shifts into the tetragonal phase at this temperature. The t-phase remains unchanged up to 2370ºC whereupon it transitions to the c-phase. Zirconia’s c-phase is then maintained up to its melting point of 2680ºC (7). The t-phase of zirconia can be secured at room temperature via the addition of certain oxide elements that possess stabilizing functions. The most common of these elements is yttrium oxide (Y2O3) (7). Tetragonal to monoclinic transformation may be triggered by external factors, such as force or temperature, and results in an expansion of 4-5% (8), inducing compressive stress that resists the propagation of cracks; a process known as “transformation toughening” (6). Zirconia can be fabricated by two different computer-assisted design and computer-assisted manufacturing (CAD/CAM) techniques. One is “soft machining” and this systems approach uses pre-sintered blocks, whereby the machine fabricates over-dimensional material frameworks in a so-called “green state”. The enlarged pre-sintered zirconia are then sintered resulting in 20-25% shrinkage to their final dimensions. The alternative technique is “hard machining”, whereby the system shapes fully sintered blocks without any dimensional changes being caused by shrinkage (9).

To emulate natural teeth, zirconia was used for substructures that were then veneered with feldspathic porcelain; however, chipping or delamination of the veneering ceramic was a major complication (10-12). To overcome this issue, monolithic zirconia was then developed. Such restorations are not suitably esthetic due to less light transmission leading to material opacity. As human color perception seems to be individual and subjective, the application of the Commission Internationale de l’Eclairage (CIE) system was popularized to determine color difference (∆E) by measuring the spectral reflectance generated from light scattering on a surface (13). The ∆E indicated “*clinically indistinguishable*” as ∆E<3, “clinically acceptable” as ∆E=3-5, and “*clinically unacceptable*” as ∆E>5. These terms appear to be objective and practical (14). Notably, it was found that color differences cannot be perceived by the human eye when ∆E has a value of less than 3.7 (15). In terms of color perception, translucency, contrast, and opalescence are essential parameters for tooth-restoration color-matching and selection (16). The state between absolute opacity and transparency is defined as “translucency”. The greater the amount of light transmitted through a material, the more its translucency is detectable (17). Translucency is affected by numerous factors such as grain size and density, type of crystalline structure and content, pigment, opaque, and distribution (including number and size) of porosity or oxygen vacancy (18,19). Translucency is generally evaluated by the translucency parameter (TP) and contrast ratio (CR) (20,21). The TP corresponds with the visual assessment of translucency. The greater the TP values, the higher the translucency of the restoration. CR ranges from 0 to 1 and negatively correlates with TP (18). “Opalescence” is the phenomenon by which a material emits a bluish hue when reflecting light and an orange/brown when transmitting it. In the fabrication of esthetic zone restorations, opalescence is concerned with closely mimicking natural appearance of dental structures (22). This optical property is determined by the opalescence parameter (OP).

Some alterations, such as to the fabrication processes or sintering temperature, including the addition of color modifiers, have been applied to improve the translucency and esthetics of zirconia and ceramics. These modifications not only affect the optical but also mechanical properties of materials (23). Crystalline content and structure, as well as pore distribution have proven to be affected by sintering parameters (24-26). Zirconia becomes translucent as the grain size enlarges but more susceptible to transformation from the t- to the m- phase (termed “spontaneous transformation”), resulting in a gradual decline in the material’s strength (27). Yttria-stabilized tetragonal zirconia polycrystalline (Y-TZP) is mainly composed of polycrystalline contents of non-homogenous crystal structures and differing refractive indexes. It commonly exhibits excessive scattering and diffuse reflectance, thereby possessing the quality of opacity (28,29). Increasing sintering temperature of monolithic zirconia leads to improved translucency with minimal effect on its flexural strength (1). Prolonging the sintering time of Y-TZP significantly enhances its optical properties by increasing the grain size and causing it to shift from the t- to the m- phase (16). The effects of heating rates and holding times on optical properties have mainly been reported in sintering temperature modifications whilst there is a lack of information regarding the effects of thermal tempering process. As such, the purpose of this study was to investigate the effects of thermal tempering process on the color characteristics of monolithic CAD-CAM ceramic materials in the sintering process. The null hypotheses were: 1) different monolithic ceramic materials would not influence the color characteristics of restorations; 2) varied thermal tempering processes would not influence the color characteristics of restorations; and 3) The interactions of different monolithic materials and thermal tempering processes would not influence the color characteristics of restorations.

## Material and Methods

-Preparation ceramic specimens

The three monolithic CAD-CAM ceramic blocks of shade A2, including partially sintered Y-TZP (I: inCoris TZI, Sirona, Bensheim, Germany), zirconia-reinforced lithium silicate glass ceramics (V: Vita Suprinity, VITA, Bad Säckingen, Germany), and lithium disilicate glass ceramics (E: IPS e.max CAD, Ivoclar Vivadent, Schaan, Liechtenstein) were prepared in bar shapes (n=45/type of material) using a diamond-coated wheel (Isomet® 1000, Beuhler, Lake Buff, IL, USA). The bar specimens were ground with silicon carbide abrasive paper # 200, 400, 600, 800, 1000 and 1200 and then polished by 1 µm diamond suspension using a polishing machine (Ecomet®3 polisher, Beuhler, Lake Bluff, IL, USA) to achieve the desired dimension (4mm width (W), 14 mm length (L) and 1.2 mm thickness (T). The inCoris TZI bar specimens were then cut to oversized dimensions (WxLxT=5x17.5x1.5 mm) to compensate for the 20% shrinkage after sintering. All specimens were cleaned in distilled water for 15 minutes using an ultrasonic cleanser (Vitasonic II, Vita Zahnfabrik, Germany), and then allowed to dry at room temperature for 60 minutes. Each material was randomly categorized into one of three groups (n=15/group) according to different thermal tempering proceses through three different cooling modes: slow cooling (S, 5°C/min), normal cooling (N, 25°C/min), and fast cooling (F, 50°C/min). The I-specimens were fired with inFire HTC furnace (Sirona Dental Systems GmbH, Bensheim, Germany) while the V- and E- specimens were fired in Programat P-310 furnace (Ivoclar Vivadent, Schaan, Leichtenstein).

-Determination color characteristics

All specimens were measured using a spectrophotometer (ColorQuest® XE, Hunter Associates Laboratory Inc., Reston, VA, USA). The device settings were fixed at a ten degrees observer angle, UV 100%, illuminant D65 as the standard wavelength between 380 nm and 780 nm and at aperture diameter of 4 millimeters. The Commission International de I’Eclairage (CIE) system using CIEL*a*b* were determined for each sample. The spectrophotometer was calibrated with a standard white tile prior to carrying out the measurements and a clear plastic jig was performed in order to maintain the position of each specimen. The L*, a*, and b* data were calculated for color appearance (∆Ew) translucency parameter (TP), contrast ratio (CR) and opalescense parameter (OP) (18).

The color appearance (∆Ew) was calculated from the differences in lightness (∆L*), green-red (∆a*), and blue-yellow (∆b*) coordinates against a white background according to equation (1).

The TP values were obtained by calculating the color differences against standard black [(B), CIE L* = 10.4, a* = 0.4, b* = 0.6] and standard white [(W), CIE L* = 96.7, a* = 0.1, b* = 0.2] backgrounds, according to equation (2).

The spectral reflectance [Y, luminance upon Tristimulus color space] was calculated from L* values, presented in equation 3. The specified white stimulus indicated perfect reflecting diffuser, and normalized by a common factor to derive for Yn that equaled to 100 (18). The Y values of specimens that were measured upon black (YB) and white (Yw) backgrounds were used to calculate for CR according to equation 4.

The OP values were determined from a* and b* coordinates that were recorded from upon a black (B) and a white (W) backgrounds using Equation (5). 

-Determination grain size

The V and E specimens were etched with 5% hydrofluoric acid for one minute to eliminate any glassy content. All the specimens were then labeled and coated with gold at a current of 10 mA and a vacuum of 130 m torr for three minutes, then dried in a desiccator cabinet. Finally, the surface topographies were evaluated under a scanning electron microscope (SEM, Hitachi S-300N, Osaka, Japan).

-Determination phase transformation

The crystalline phases of ceramic materials were determined by their relative proportion of microstructures using an X-ray diffractometer (XRD, PANalytical, Empyrean, Almelo, Netherlands). The specimens were scanned at a diffraction angle (2θ degree) of 0–40º with a 0.02º step size at two-second intervals using copper k-alpha (Cu Kα) radiation. Each phase was analyzed by cross-reference with the Joint Committee of Powder Diffraction Standards database. The ratio of m- to t- phases was measured by the intensity of peaks using X’Pert Plus software (Philips, Almelo, Netherlands) and the Garvie-Nicholson formula was used to calculate the fraction of m- phase to the entire phase (Xm) as shown in equation 6-8 (30).

Where: Im and It are the integral intensities of monoclinic and tetragonal phases

*C is a composition-dependent correction factor (C = 1.32)*.

Xt is the Toraya-corrected mass fraction of tetragonal zirconia.

-Statistical analysis

Two-way ANOVA and Bonferroni Post Hoc using IBM SPSS for Windows 20 (SPSS Inc., Chicago, IL, USA) were used to determine the significance differences in color parameters of monolithic materials subjected to different cooling rates in the sintering process. A result was considered statistically significant at *p*< 0.05. Descriptive analysis was applied to determine the color characteristics, grain size, and phase transformation of monolithic materials.

## Results

**Figure 1:**
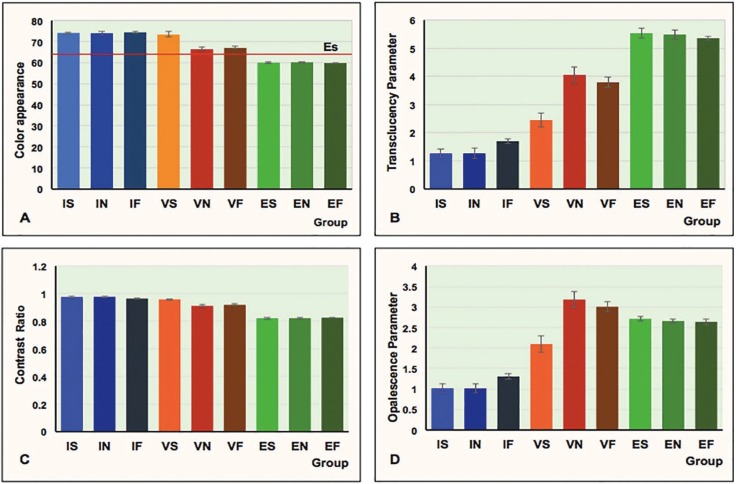
Color appearance (A), translucency parameter (B), contrast ratio (C), and opalescence parameter (D) of of inCoris TZI (I), Vita Suprinity (V), IPS e.max CAD (E) upon slow- (S), normal- (N), and fast- (F) thermal tempering processes.

The mean, standard deviation (SD), and 95-% confidence interval of the color parameters (appearance, translucency parameter, contrast ratio, and opalescence parameter) for each group are illustrated in  Table 1 and Figure 1. The mean±sd of ΔEW, TP, CR, and OP were 74.15±0.46, 1.26±0.15, 0.977±0.006, and 1.02±0.12 for IS; 74.00±0.83, 1.27±0.19, 0.977±0.007, and 1.02±0.12 for IN; 74.44±0.64, 1.70±0.08, 0.965±0.003, and 1.30±0.07 for IF; 73.35±1.32, 2.44±0.24, 0.958±0.006, and 2.10±0.20 for VS; 66.37±0.88, 4.05±0.3, 0.911±0.010, and 3.18±0.20 for VN; 67.02±0.65, 3.79±0.17, 0.919±0.006, and 3.01±0.13 for VF; 60.01±0.30, 5.53±0.17, 0.821±0.006, and 2.71±0.06 for ES; 60.18±0.23, 5.49±0.17, 0.822±0.006, and 2.66±0.05 for EN; and 59.82±0.26, 5.36±0.06, 0.826±0.002, and 2.64±0.07 for EF.

**Table 1:**
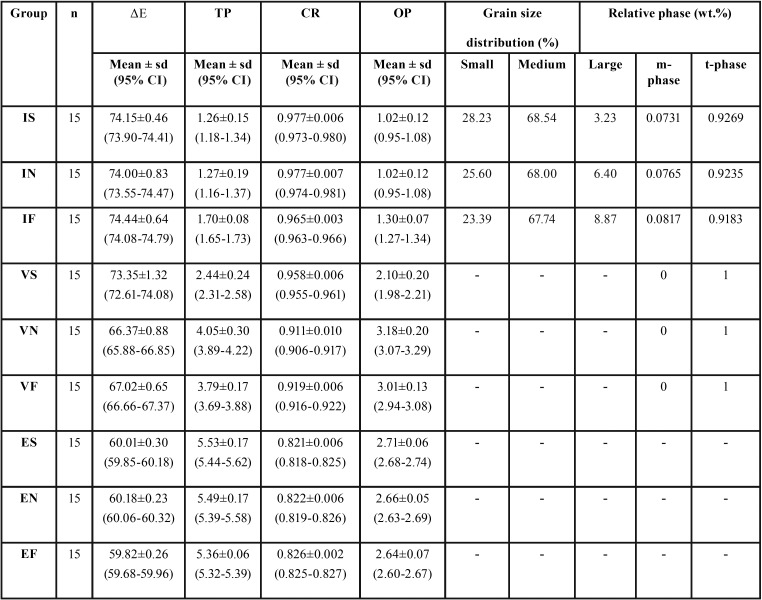
Mean, standard deviation (sd), 95% confidential interval (CI), color appearance (ΔΕ), translucency parameter (TP), contrast ratio (CR), and opalescent parameter (OP), grain size distribution (%), and relative phase content (wt.%) of inCoris TZI (I), Vita Suprinity (V), IPS e.max CAD (E) upon slow- (S), normal- (N), and fast- (F) thermal tempering processes.

Two-way ANOVA indicated a statistically significant difference in ΔEW, TP, CR, and OP due to differing materials, tempering processes, and interactions (*p*<0.05), as shown in  Table 2. Post-hoc Bonferroni multiple comparisons demonstrated that different monolithic materials possessed significant differences (*p*<0.05) in ΔEW, TP, CR, and OP, as presented in  Table 3. Post-hoc Bonferroni multiple comparisons demonstrated that different tempering process had significant differences (*p*<0.05) in ΔEW,, TP, CR, and OP, except between N-F, as presented in  Table 3. Post-hoc Bonferroni multiple comparisons demonstrated that a combination of different monolithic materials and different cooling rates produced significant differences in the ΔEW, value (*P*<0.05), except between IS-IN, IS-IF, IS-VS, IN-IF, IN-VS, VN-VF, ES-EN, ES-EF, and EN-EF (*P*>0.05), as well as significant differences in the TP value (*P*<0.05), except for IS-IN, ES-EN, ES-EF, and EN-EF group (*P*>0.05), and significant difference in CR value (*P*<0.05) except for the IS-IN, IF-VS, ES-EN, ES-EF, and EN-EF groups (*P*>0.05), and further significant differences in the OP value (*P*<0.05), except for the IS-IN, ES-EN, ES-EF, and EN-EF groups (*P*>0.05), as presented in  Table 4 and Figure 1.

**Table 2:**
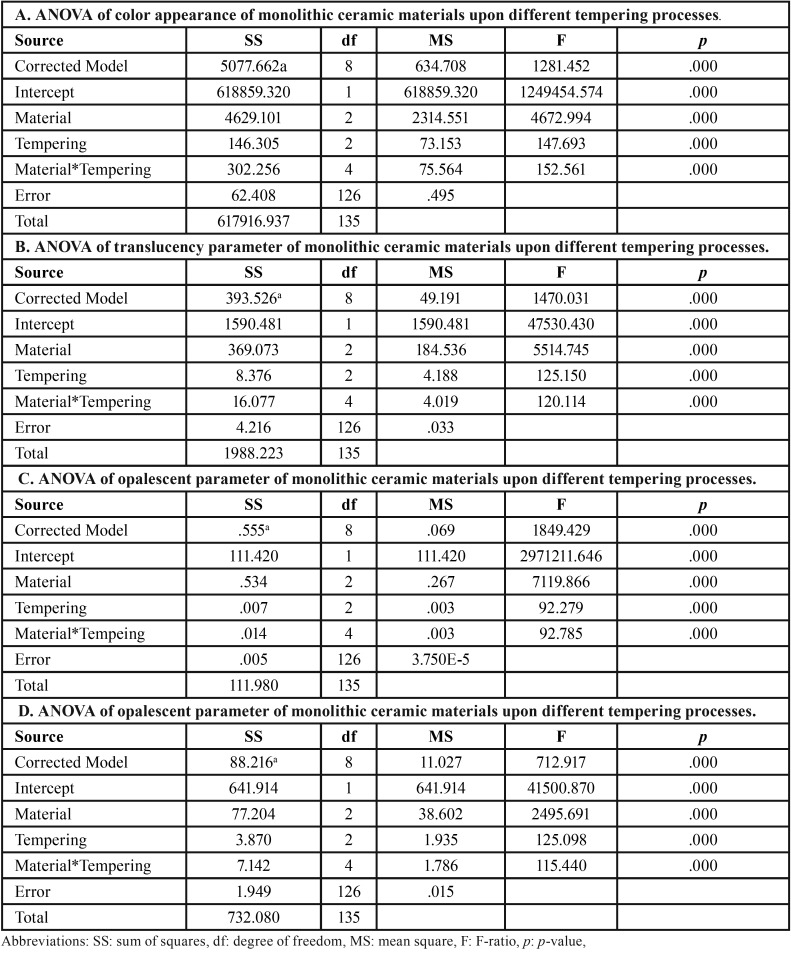
An analysis of variance (ANOVA) of color appearance (A), translucency parameter (B), contrast ratio (C), and opalescent parameter (D) of three monolithic ceramic materials upon different thermal tempering processes.

**Table 3:**
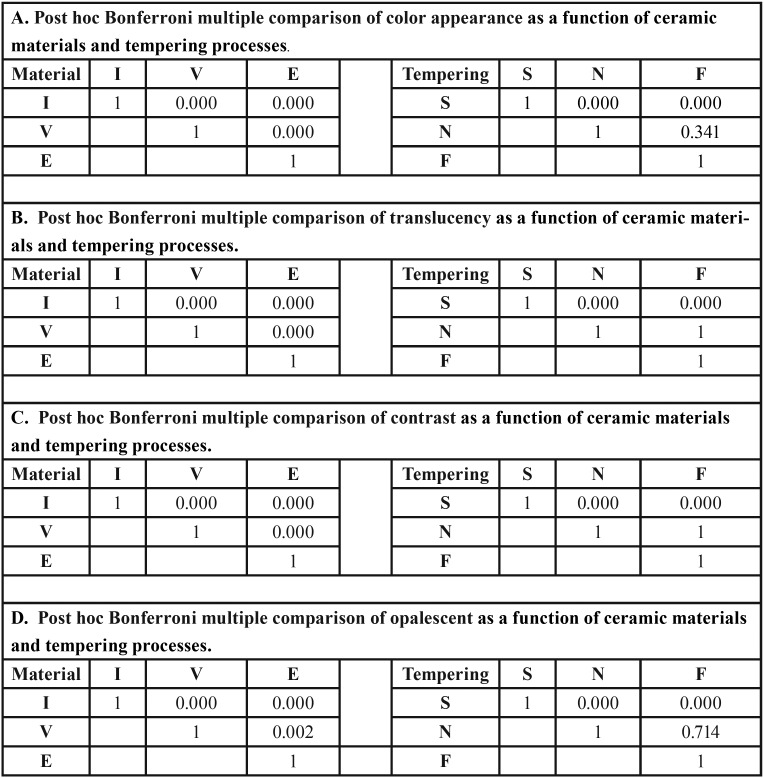
Post hoc Bonferroni multiple comparisons of color appearance (A), translucency parameter (B), contrast ratio (C), and opalescent parameter (D) of inCoris TZI (I), Vita Suprinity (V), IPS e.max CAD (E) upon slow- (S), normal- (N), and fast- (F) thermal tempering processes.

**Table 4:**
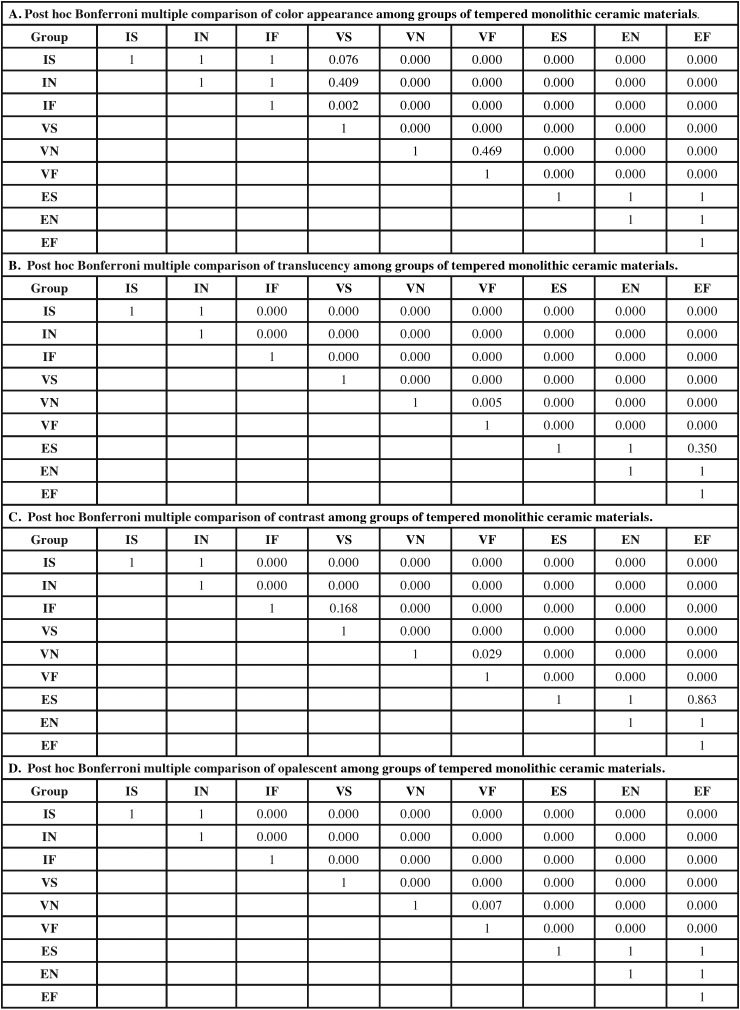
Post hoc Bonferroni multiple comparisons of color appearance (A), translucency parameter (B), contrast ratio (C), and opalescent parameter (D) of inCoris TZI (I), Vita Suprinity (V), IPS e.max CAD (E) upon slow- (S), normal- (N), and fast- (F) thermal tempering process among groups.

**Figure 2:**
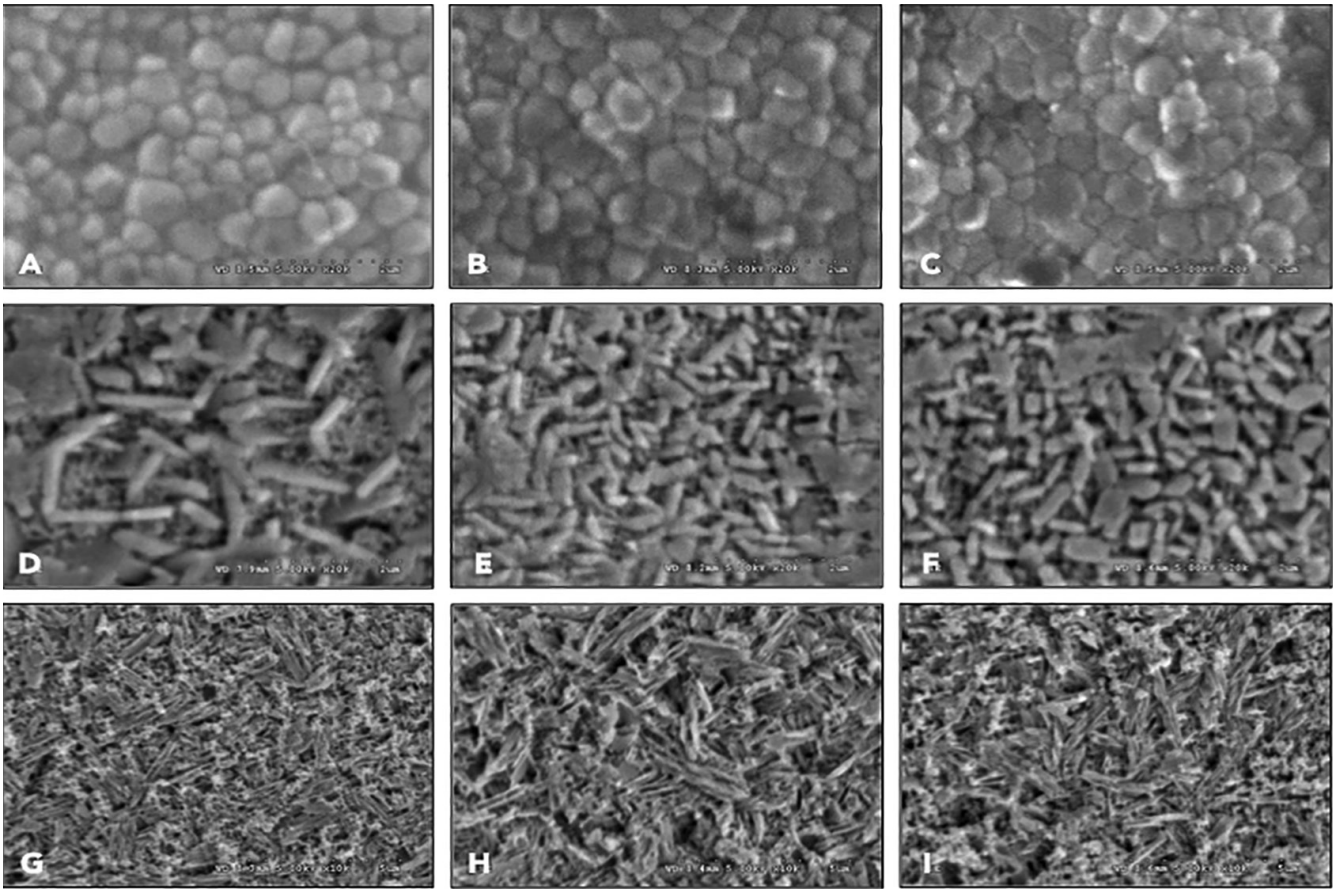
SEM photomicrographs indicated grain size and grain distribution of monolithic ceramic material for of inCoris TZI (A, B, C), Vita Suprinity (D, E, F), IPS e.max CAD (G, H, I) upon slow- (A, D, G), normal- (B, E, H), and fast- (C, F, I) thermal tempering processes, at X20K magnification, except for G, H, I at X10K magnification.

The microscopic structures of inCoris TZI, Vita Suprinity, and IPS e.max CAD are shown in Figure 2. With respect to the inCoris TZI group, the crystal structures were defined according to three grain sizes: small (0.1-0.38 μm), medium (0.39-0.66 μm), and large (0.67-0.93 μm). All inCoris TZI groups indicated crystal structures mostly of medium grains. Fast tempering procedure resulted in grain growth, and displayed an increase in medium and large grain size, more so than at slow and normal tempering procedure. The average grain size (µm) of IS, IN, and IF were 0.460, 0.470, and 0.482, respectively. The quantities (%) of small, medium, and large grain sizes were 28.23, 68.54, and 3.23 for IS; 25.60, 68.00, and 6.40, for IN; and 23.39, 67.74, and 8.87 for IF, as shown in Table 1. Additionally, the SEM indicated a defective integration of crystal structures at the grain boundaries in the slow and normal tempering groups.

Vita Suprinity exhibited a roundish needle-like crystal structure of lithium disilicate, and grain of zirconia. The average lithium disilicate grain sizes (µm) for VS, VN, and VF were 0.873, 0.601, and 0.609, respectively. Furthermore, the VN and VF crystals were observed to be more intense and of less porous than those of VS. IPS E.max CAD showed a needle-like crystal structure of lithium disilicate. The average lithium disilicate grain sizes (µm) for ES, EN, and EF were 1.724, 1.707, and 1.664, respectively. The SEM of ES, EN, and EF showed no differences in grain density and porosity.

The microstructure analysis of the specimens using XRD was shown in  Table 1. In the inCoris TZI group, the XRD patterns demonstrated a great amount of tetragonal phase with a small amount of m-phase. The t-phase was detected at the diffraction angles (2θ degree) of 30.20°, 34.63°, and 35.20° for IS, and IN; and 30.11°, 34.53°, and 35.09° for IF. The monoclinic phases were detected at 27.79° and 31.12° in all inCoris TZI groups. All the data corresponded to the crystallographic patterns of each specimen, as indicated by the XRD standard file. The relative concentrations (wt.%) of monoclinic phases in relation to the total number of zirconia phases revealed variations in the amount of the transformation from the tetragonal to the monoclinic phase, owing to firing parameters, as presented in  Table 1. The relative weight percentage (wt.%) concentrations of the monoclinic and tetragonal phases were 0.0731 and 0.9269 for IS; 0.0765 and 0.9235 for IN; and 0.0817 and 0.9183 for IF. The relative amount of phase content was related to the cooling rate of the sintering process. An increase in the relative amount of monoclinic content was observed when the zirconia was subjected to a fast tempering.

In the Vita Suprinity group, XRD revealed a great amount of crystal structure of lithium disilicate, followed by lithium metasilicate, lithium orthophosphate and tetragonal phase of zirconia. Lithium disilicate of VS was observed at 23.94°, 24.51°, 25.00°, and 37.81°; lithium metasilicate of VS was detected at 27.11°, 33.17°, and 38.39°; lithium orthophosphate of VS was detected at 22.37°, 23.24°, and 38.80°; and tetragonal zirconia was detected at 30.29°. Lithium disilicate of VN was observed at 23.80°, 24.36°, 24.85°, and 37.67°; lithium metasilicate of VN was detected at 26.96°, 33.04°, and 38.41°; lithium orthophosphate of VN was detected at 22.31°; and tetragonal zirconia was detected at 30.14°. Lithium disilicate of VF was observed at 23.96°, 24.52°, 25.02°, and 37.83°; lithium metasilicate of VF was detected at 27.13°, 33.20°, and 38.58°; lithium orthophosphate of VF was detected at 22.31, and 23.15°. and tetragonal zirconia was detected at 30.24°. In the IPS e.max CAD group, XRD patterns showed the majority of crystal structures were lithium disilicate and lithium metasilicate with a small number being lithium orthophosphate. Lithium disilicate of ES was observed at 23.97°, 24.53°, 25.03°, and 37.83°; lithium metasilicate of ES was detected at 38.37°; and lithium orthophosphate of ES was detected at 22.51°, 23.26°, and 34.02°. Lithium disilicate of EN was observed at 23.80, 24.37, 24.86, 37.67, and 39.30 °; lithium metasilicate of EN was detected at 38.21°; and lithium orthophosphate of EN was detected at 22.34°, 23.11°, and 36.39°. Lithium disilicate of EF was observed at 23.96°, 24.53°, 25.02°, and 37.83°; lithium metasilicate of EF was detected at 38.37°; and lithium orthophosphate of EF was detected at 22.48°, 23.28°, and 34.01°.

## Discussion

This study aimed to achieve improved optical properties by altering the tempering process in the sintering process of three monolithic materials: inCoris TZI, Vita Suprinity, and IPS e.max CAD. The modification of the sintering parameters was interesting, in terms of providing appropriate information for clinicians and laboratory technicians in order to manage the optical properties of materials and to fabricate restorations with a natural-tooth appearance, enhanced translucency, better contrast, as well as improved opalescence.

A multitude of scientists had studied and reported on alterations to the heating temperature and holding time but a lack of research had been conducted on the cooling phase. This study aimed to determine the optical characteristics (∆Ew, TP, CR, and OP) of monolithic materials by altering the tempering process. The tempering processes were classified into three categories: slow (5ºC/min), normal (25ºC/min), and fast (50ºC/min). The study herein found statistically significant differences in the color characteristics of different monolithic materials at different tempering, as well as in their interactions; therefore, all null hypotheses were rejected. As far as color appearance was concerned, the VITA classic block shade A2 was used, that possesses standard color appearance (Es) of 62.21. According to a color perceivability by CIE system, the ∆E indicated “*clinically indistinguishable*” as ∆E<3, “*clinically acceptable*” as ∆E=3-5, and “*clinically unacceptable*” as ∆E>5. 14 All IPS e.max CAD groups were considered as “*clinically indistinguishable*”; all Vita Suprinity groups were considered as “*clinically acceptable*”; whilst all inCoris TZI groups were categorized as “*clinically unacceptable*”. In addition, based on the human perception, differences in color cannot be detected by the human eye when ∆E has a value of less than 3.7 (15). Unfortunately, The color differences observed in InCoris TZI and Vita Suprinity when subjected to various sintering protocols were over 3.7. Thus, the use of IPS e.max CAD proved promising for matching to selected shades, even when the sintering procedure was modified. Lithium disilicate glass ceramic was advocated to be the gold standard restoration for esthetics (4).

In terms of translucency, this is a crucial optical parameter to simulate natural tooth appearance, perfectly match with the surrounding structure, especially in the anterior region (31) and esthetic zone. This current study showed that the translucency parameter and opalescence ratio of ES was the highest, followed by EN, EF, VN, and VF. However, the contrast ratio of ES was the lowest, followed by the others in the same sequence. This indicated that TP correlated with OP and negatively correlated with CR. The translucency of inCoris TZI was significantly enhanced upon an increase in the rate of tempering, as observed by increased TP and decreased CR values. This was likely related to the complete grain growth of zirconia crystalline structures, and resulting in enlarged grains, as well as pore- and defect reductions in the grain boundaries. Porosity may impact the optical properties of zirconia because of the different refractive indices of zirconia and air (28,29).

**Figure 3:**
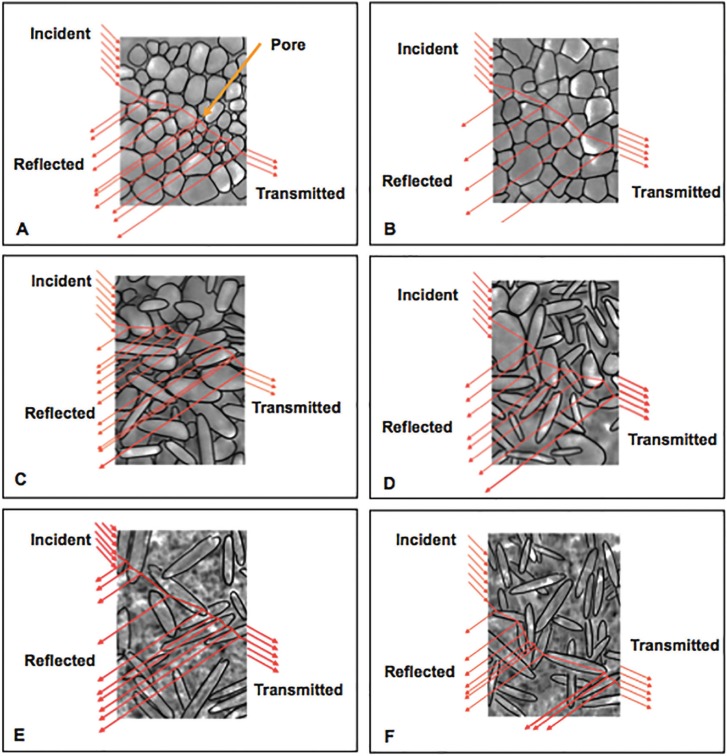
Possible explanation the behavior of light in reflection, scattering, and transmission in relation with microstructure for inCoris TZI (A, B), Vita Suprinity (C, D), IPS e.max CAD (E, F) upon slow- (A, C, E), normal-, and fast- (B, D, F) thermal tempering processes.

The average grain size of IF was bigger than those of IS and IN, due to increased rate of tempering in the sintering process, which might be capable of reducing pores at grain boundaries by rapidly inducing stress on the materials. This led to a t-m phase transformation and also resulted in grain enlargement (16). This was supported by an XRD analysis demonstrating a shift in the crystalline content from the tetragonal to the monoclinic phase as well as grain enlargement in the zirconia, as evidenced by the SEM when increasing the rate of tempering. A combination of reducing the porosity and increasing the density of zirconia may have caused an increase in the homogeneity of its crystalline structure, which promoted better specular reflectance and optical transmission with minimized refraction, as shown in Figures 3 (A and B). As such, this study provides compelling evidence that increasing (rather than decreasing) the rate of tempering may achieve increased translucency. In the same way, the translucency of Vita Suprinity was significantly increased upon an increase in the rate of tempering. This may have been due to the grain and pore distribution, as mentioned above. The micrograph of all the zirconia-reinforced lithium silicate glass ceramic groups showed that the crystalline structures were mainly composed of lithium disilicate and minor tetragonal zirconia. The VS group illustrated longer and bigger grains, as well as larger and more numerous oxygen vacancies, resulting in less light transmission and translucency compared to the VN and VF groups shown in Figures 3 (C and D). Although a larger grain size was reported to achieve better translucency, the VS group showed less translucency than the other groups (4). This might have been because the effect of numerous and large porosities played a more important role in translucency than grain size. The grain size of VS was observed to be greater than those of the other groups but might be considered to fall within the same range as the VN and VF groups. Nevertheless, the VN group showed the highest translucency due to the small and a few porosities occupied.

IPS e.max CAD showed no statistical differences in translucency at different tempering processes. This was supported by the SEM indicated the same characteristics of grain and porosity distribution as shown in Figure 3 (E and F). According to the manufacturer’s recommendation, inCoris TZI was suggested for a normal tempering, whilst Vita Suprinity and IPS e.max CAD were suggested for slow tempering. In order to achieve the optimum translucency of each material, the study herein proposed inCoris TZI for a fast tempering , Vita Suprinity for a normal tempering, and IPS e.max CAD for a fast tempering in order to minimize the sintering time because of no statistical differences in all the color parameters of ES, EN, and EF. These results provided the data by which clinicians and laboratory technicians may optimize the optical properties of materials by altering the sintering parameters. This study supports increased rates for thermal tempering process of sintering monolithic materials to achieve better translucency. The mechanical properties of restorations resulting from a variety of materials and tempering method should be the subject of future study for clinical application.

## Conclusions

The results of this study indicated that the color appearance (∆EW), translucency parameter (TP), contrast ratio (CR), and opalescence parameter (OP) values of inCoris TZI, Vita Suprinity, and IPS e.max CAD were significantly different and were affected by the material type, the thermal tempering process, and their interaction. Increasing the thermal tempering rate of Y-TZP resulted in a bigger grain size and a t-m phase transformation leading to higher translucency. Thus, to achieve optimum translucency, a fast thermal tempering process is suggested for inCoris TZI and IPS e.max CAD, whilst a normal tempering process is recommended for Vita Suprinity.
